# Circulating Metabolites Originating from Gut Microbiota Control Endothelial Cell Function

**DOI:** 10.3390/molecules24213992

**Published:** 2019-11-05

**Authors:** Amedeo Amedei, Lucia Morbidelli

**Affiliations:** 1 Department of Experimental and Clinical Medicine, University of Florence, Viale Pieraccini 6, 50134 Florence, Italy; aamedei@unifi.it; 2 Department of Life Sciences, University of Siena, Via A. Moro 2, 53100 Siena, Italy

**Keywords:** gut microbiota, metabolite, endothelial cell, endothelial dysfunction, cardiovascular diseases, hypertension, nitric oxide, inflammation, reactive oxygen species, polyphenols

## Abstract

Cardiovascular functionality strictly depends on endothelial cell trophism and proper biochemical function. Any condition (environmental, pharmacological/toxicological, physical, or neuro-humoral) that changes the vascular endothelium has great consequences for the organism’s wellness and on the outcome and evolution of severe cardiovascular pathologies. Thus, knowledge of the mechanisms, both endogenous and external, that affect endothelial dysfunction is pivotal to preventing and treating these disorders. In recent decades, significant attention has been focused on gut microbiota and how these symbiotic microorganisms can influence host health and disease development. Indeed, dysbiosis has been reported to be at the base of a range of different pathologies, including pathologies of the cardiovascular system. The study of the mechanism underlying this relationship has led to the identification of a series of metabolites (released by gut bacteria) that exert different effects on all the components of the vascular system, and in particular on endothelial cells. The imbalance of factors promoting or blunting endothelial cell viability and function and angiogenesis seems to be a potential target for the development of new therapeutic interventions. This review highlights the circulating factors identified to date, either directly produced by gut microbes or resulting from the metabolism of diet derivatives as polyphenols.

## 1. Vascular Endothelium and Its Functions

**Figure 1:**
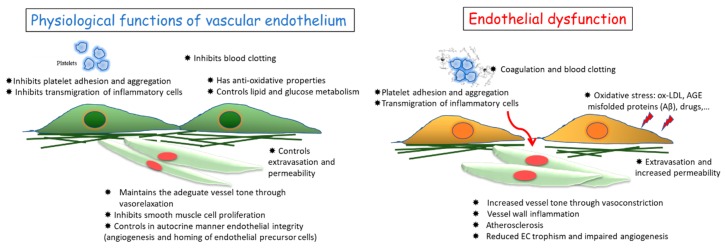
Features and functions of a healthy endothelium (**left panel**) and dysfunctional endothelium (**right panel**). The evolution from functional to dysfunctional endothelial cells (ECs) depends on various factors both exogenously (pollutants, toxins, drugs, diet components) and endogenously produced (oxidized lipoproteins: ox-LDL, hyperglycemia and glycation products: AGE, misfolded proteins such as beta-amyloid-Aβ, reactive oxygen species: ROS). Here, we have focused on the hypothesis that microbiota derived metabolites can influence endothelial behavior and thus cardiovascular disease (CVD) risk and manifestation.

A healthy endothelium, the cell monolayer which covers the inner part of blood vessels, is crucial for the homeostasis of the cardiovascular system and whole body functioning [1,2]. First, it regulates blood pressure, playing a protective role against hypertension and atherosclerosis, which in the long term can cause fatal accidents. Additionally, it sustains the function of the vessel wall as a blood barrier, preventing infiltration of leukocytes and inflammatory processes into the vascular wall and surrounding tissues [1,2]. In physiological conditions, endothelial cells (ECs) produce and release anti-aggregatory and anti-coagulative mediators that prevent vascular stenosis and thrombus formation [1,2]. Altogether, mature ECs, endothelial progenitor cells, and circulating ECs contribute to the physiological maintenance of cardiovascular functions such as vessel tone, permeability and intima thickness, vascular remodeling, and angiogenesis (Figure 1, left panel). 

Conversely, endothelial dysfunction has been identified as a hallmark of the majority of cardiovascular diseases (CVD), including atherothrombosis, pulmonary hypertension, microangiopathies associated with neurodegenerative diseases, diabetes, liver steatosis, sepsis, and cancer metastasis [3]. Various conditions can be responsible for endothelial dysfunction, such as turbulent blood flow, shear stress, hyperglycemia, hypoxia, ageing, hypercholesterolemia, and hypertension [4,5]. Dysfunction of ECs is correlated with an altered production of key regulators of vascular homeostasis, such as nitric oxide (NO) and growth factors, and/or impaired activity (uncoupling) of endothelial NO synthase (eNOS), associated with increased levels of reactive oxygen species (ROS) and vascular oxidative status [6]. Inflammatory factors, for example interleukin-1 (IL-1), interleukin-6 (IL-6), tumor necrosis factor alpha-α (TNF-α), intercellular adhesion molecule 1 (ICAM-1), and loss of antioxidant mechanism are among the most important molecular determinants of vascular dysfunction [2,5] (Figure 1, right panel). 

Accordingly, when endothelial dysfunction occurs, the endothelium’s ability to perform one or all of its functions is decreased, switching to a pro-inflammatory profile, discontinuation of endothelial monolayer, pathological angiogenesis, and decreased vasodilation, together with pro-thrombotic properties [2,5]. Our studies and those of others have demonstrated that endothelial dysfunction is associated with impairment of angiogenic processes and the subsequent pathological remodeling of the microcirculation that contributes to the onset of various diseases [7,8,9]. 

At the vascular level, age-related low-grade, chronic, and systemic inflammation is indicated by the term “inflammaging”, while the imbalance of the immune response leads to immunosenescence. Both phenomena severely influence vessel cellular components in their functionality and viability [10]. Similar to natural ageing is the condition of unloading or gravity absence, as in astronauts, where endothelial features resemble endothelial dysfunction and angiogenesis impairment [11,12].

Indeed, endothelial function has been proposed as a “barometer for cardiovascular risk”. In this scenario, several conditions, such as hypertension, diabetes, and atherosclerosis, and lifestyle choices such as smoking, high-fat diet, alcohol consumption, and physical inactivity are risk factors for endothelial dysfunction. It is of note that even if age remains the main unchangeable determinant of vascular age, healthy vascular aging can be achieved, and progress has been made in the maintenance of heathy vasculature, related to both lifestyle and diet and drug therapies. 

However, preventive or therapeutic treatments to maintain endothelial functionality and integrity are real medical needs. Since the endothelium is a regulator of exchanges between the vascular wall and surrounding tissues, it is not surprising that (i) vascular endothelium can be sensitive to endogenous mediators (including microbiota derivatives) and (ii) dysfunctional ECs can lead to the impairment of other tissues. 

### 1.1. Physiological and Pathological Angiogenesis

Angiogenesis, the growth of new capillaries from the pre-existing blood vessels, is primarily induced by hypoxia in growing, remodeling, or ischemic tissues, when there are oxygen and nutrient demands. This neovascular growth involves EC proliferation, migration, and functional differentiation [13,14]. 

In physiology, angiogenesis plays a fundamental function in placenta establishment, embryonic development, wound repair, and tissue remodeling and engineering [13]. Indeed, angiogenesis, occurring after vasculogenesis, is essential for embryonic growth, since it is required for the proper development of a functional circulatory system, delivering nutrients and oxygen to every cell of the body and allowing catabolite disposal [15]. In contrast, angiogenesis in the adult organism is restricted to a few processes, specifically those related to the reproductive cycle, wound healing, and bone repair. In all these events, angiogenesis is finely controlled by the balance between pro- and anti-angiogenic molecules and occurs transiently, although, when dysregulated, the formation of new blood vessels may contribute to various diseases (oncogenic, ischemic, inflammatory, and infective ones) [16,17]. The long list of angiogenesis-dependent disorders comprises a number of apparently unrelated diseases, including proliferative retinopathies, rheumatoid arthritis, psoriasis, endometriosis, and many types of tumors. A common feature of the angiogenesis-dependent diseases is the so-called angiogenic switch, defined as an imbalance of pro-angiogenic regulatory factors over anti-angiogenic ones [13]. Conversely, there are pathologies such as ischemic diseases where there is an insufficient blood supply, and tissue neovascularization is needed. In principle, compounds able to control the angiogenic balance could be good candidates for the pharmacological treatment of angiogenesis-dependent disorders (anti-angiogenic compounds), or for those diseases with a deficient blood supply (pro-angiogenic strategies). These needs explain the great interest in angiogenesis from pharmaceutical companies and academic groups searching for strategies and compounds able to affect (positively or negatively) angiogenesis [18,19,20,21,22,23].

### 1.2. Molecular Regulation of Angiogenesis

As previously reported, neovascular growth involves endothelial proliferation, migration, and differentiation under stimulation by specific angiogenic inducers that activate defined molecular pathways, biochemical cascades, and genomic changes. Firstly, pro-angiogenic growth factors bind to their receptors in ECs, which are activated to release matrix metalloproteinases (MMPs). These proteases degrade the basement membrane, allowing the activated ECs to migrate and proliferate outside of the preexisting blood vessel. Then, with the participation of adhesion molecules (e.g., integrin αvβ3 and αvβ5) and MMPs, the nascent vessel spreads. The new vessel sprouts connect to each other to produce a tube or a loop and, finally, pericytes merge in the vascular wall to stabilize the newly formed vessels [17]. 

Different factors can promote or inhibit angiogenesis. All these molecules, with their opposing effects, control angiogenesis in a balanced manner, and a change in this equilibrium can lead to pro- or anti-angiogenic outcomes. Epithelial or stromal cells, inflammatory elements, and neoplastic and other diseased cells produce and release angio-modulatory factors in response to inflammation, hypoxia, and other pathophysiological settings [24].

The key growth factor upregulated in these various conditions is vascular endothelial growth factor (VEGF), which has a high specificity for the vascular endothelium. The expression of its receptor (VEGFR) is crucial for EC activation. Other growth factors contribute to vascular development and promote angiogenesis, such as fibroblast growth factor-1 and -2, platelet-derived growth factor, transforming growth factor-β, and the angiopoietins and their tyrosine kinase receptors, expressed in stromal and vascular cells. When inflammation is the trigger of angiogenesis outcomes, other mediators are involved, in particular cytokines [24], prostaglandins [25], and NO [26,27], which mediate and amplify the angiogenic activity of growth factors [28,29,30]. Conversely, some chemokines negatively control angiogenesis, together with known anti-angiogenic molecules, such as endostatin, angiostatin, thrombospondins, and pigment epithelium-derived factor [31].

The research exploring targets and molecules able to maintain EC physiological function and modulate or promote angiogenesis is ongoing. Our attention has been focused on natural products as nutraceuticals, since they promise to be a good source for prevention and therapeutic approaches in most of disorders associated with aberrant or non-functional vessels [32,33].

Beside exogenous derivatives, we can hypothesize that there are endogenously produced metabolites in circulation able to influence endothelial cell behavior. Among them, particular attention in this review has been paid to metabolites derived from gut microbiota (GM) metabolism.

## 2. Gut Microbiota (GM) Influence on Disorders Characterized by Endothelial Dysfunction

The GM is an assorted ecosystem that comprises bacteria, protozoa, archaea, viruses, and fungi, which are in a specific symbiosis with each other and the human body. It is now known that GM plays important roles in physiological conditions of human health (participating in digestion, immunomodulation, and cardiovascular system performance), and also in different pathological disorders.

The healthy GM consists mainly of Gram-positive Firmicutes, Gram-negative Bacteroidetes, and Gram-positive Actinobacteria, and dysbiosis (dysregulation of GM composition) leads not only to gut related diseases, such as inflammatory bowel disease (IBD) and colorectal cancer [34], but also to systemic disorders such as obesity [35], allergies, diabetes mellitus, and CVD [36,37]. In particular, comparing GM fingerprints between CVD patients and healthy controls has shown that the diversity of beneficial bacteria is reduced in CVD patients [38], suggesting that the GM role in CVD is significant. Varied bacterial types are involved in different forms of CVD, including arterial thrombosis (Allobaculum, *Candidatus arthromitus*, and Lachnospiraceae), coronary artery disease (*Helicobacter pylori*) and atherosclerosis (Clostridiaceae, Clostridales, and Ruminococcus). Li et al. [39] demonstrated that abundant mucin-degrading bacterium (*Akkermansia muciniphila*) showed various beneficial effects on metabolism. An eight-week treatment with *A. muciniphila* significantly decreased atherosclerotic plaque development without any impact on hypercholesterolemia.

Finally, it was well reviewed in reference [40] that other major bacteria, such as *Tyzzerella 4*, *Tyzzerella*, *Catenibacterium*, *Alloprevotella*, *Prevotella 2*, and *Prevotella 7*, have a documented role in CVD.

Similarly, gut dysbiosis has been reported in astronauts and experimental animals maintained in unloading conditions. Indeed, both microgravity and space radiation predispose to different space related disorders, most being accompanied by immune system dysregulation [41,42]. In fact, in healthy conditions, the GM plays a crucial role in the maintenance of the host’s physiological conditions by modulating its immunity. The GM can modulate the functionality of neutrophils, as well as T-cell differentiation into the different subpopulations (Th1, Th2, and Th17 or Treg) [43]. In addition, by fermenting non-digestible complex carbohydrates, GM bacteria can secrete short-chain fatty acids (SCFAs) which can cross the intestinal epithelium and reach the lamina propria, directly influencing mucosal immune responses [44].

Gut bacteria may affect the circulatory system through two main pathways. First, gut bacteria and/or their metabolites may stimulate the enteric nervous system, which in its turn can affect the activity of the brain centers controlling the cardiovascular system, as already described for cytokines [45,46,47]. Second, gut bacteria and their metabolites may pass into the circulation through gut–blood barrier disruption and then affect the function of the tissues and organs that control circulatory system homeostasis, such as the blood vessel wall, heart, and blood cells. The proper functioning of the gut–blood barrier may be altered in various diseases, including dysbiosis. Thus, easier access of gut-derived molecules to the circulation may influence the evolution of underlying disease, this vicious cycle being deleterious for the entire organism.

Consequently, normalizing gut microbial composition and metabolism has been proposed as a strategy to reverse diet- and environment-induced vascular dysfunction [37].

## 3. Influence of Gut-Derived Metabolites on Vascular Endothelium and the Cardiovascular System

Gut microorganisms are involved in the synthesis of a plethora of bioactive compounds contributing to normal physiology or prompting diseases [48,49].

The chemical composition of the GM includes amino acids, lipids, sugars, biogenic amines, organic acids, peptides and proteins, glycolipids, oligosaccharides, terpenoids or secondary bile products, and volatile small molecules [50,51,52].

**Figure 2:**
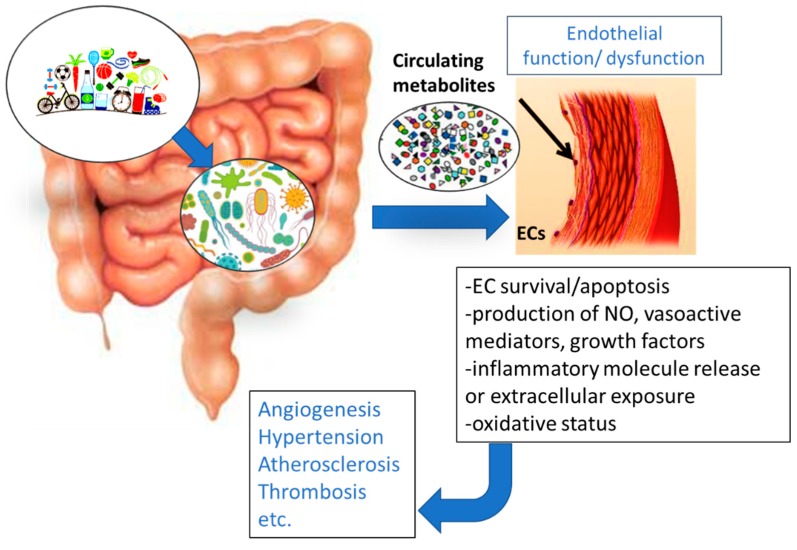
Schematic representation of the activity of circulating metabolites originating from the gut microbiota (GM) on endothelial cell function. Alteration of endothelial viability and metabolism in its turn influences the outcome of hypertension, atherosclerosis, and other CVDs, and the type and extent of the angiogenic response.

For CVD in particular, the association with the GM and its metabolites has been recently reported [48,53,54,55]. In this review, we have focused on GM-derived metabolites and their definite influence on endothelial function and CVD (Figure 2). 

First, we describe the deleterious metabolites, followed by the beneficial ones and those with controversial results.

### 3.1. Trimethylamine N Oxide (TMAO)

The GM can cleave dietary compounds containing trimethylamine for the release of trimethylamine (TMA), which can be further oxidized by liver flavin monooxygenase (FMO) to trimethylamine N oxide (TMAO), and is then excreted in the urine [48,54]. The precursors from which GM organisms synthetize TMA include a series of molecules abundant in diets rich in animal products as choline, phosphatidylcholine, glycerophosphocholine, carnitine, betaine and its derivatives, and TMAO [54,56].

Several studies have demonstrated the pro-atherogenic properties of TMAO. Plasmatic TMAO levels are associated with CVD prevalence and, when normalized for conventional cardiac risk factors and renal dysfunction, can independently predict myocardial infarction, stroke, or death [48,53,55]. Mechanistically, platelets, vascular smooth muscles, and ECs are influenced by TMAO. In vitro experiments and animal models have documented the prothrombotic effect of TMAO by enhancing platelet aggregation [57]. Oral supplementation with choline consistently increases TMAO levels and platelet aggregation [58]. TMAO activates vascular smooth muscle cell mitogen activated protein kinase (MAPK) and EC dysfunction through nuclear factor-κB (NF-κB) signaling, responsible for upregulation of inflammatory signals and adhesion of leukocytes to ECs [59]. Reduced EC repair by TMAO has also been reported in in vitro studies [60]. Recently, it has been reported that enhanced plasma TMAO levels were associated with reduced circulating endothelial precursors, endothelial dysfunction, and more severe cardiovascular events [61].

By means of metabolomic analysis, various microbial metabolites have been identified as predictors of coronary heart disease [62]. These metabolites included 15 exclusive choline metabolites, GlcNAc-6-P, and mannitol. Combination of metagenomic and metabolomics data revealed the formation of GlcNAc-6-P by *Clostridium* sp. HGF2, and production of mannitol by *Clostridium* sp. HGF2, *Streptococcus* sp. M143, and *Streptococcus* sp. M334 [62]. However, the exact mechanism by which coronary disease development is influenced by these bacterial metabolites remains to be elucidated. 

The strategies to control TMAO levels encompass the development of enzymatic inhibitors of GM enzymes (Table 1). The molecule 3,3-dimethylbutanol (DMB) has been designed as a choline analogue to inhibit choline TMA lyase. A decrease in circulating TMAO has been reported, attenuating the atherogenic effect of choline [56].

Additionally and intriguingly, the phytoalexin resveratrol, belonging to the polyphenol stilbenoids group abundantly present in grape skin and seeds, can decrease plasma TMAO and subsequent atherosclerosis in ApoE^−/−^ mice. GM remodeling, with increased levels of the genera *Lactobacillus* and *Bifidobacterium,* was associated with increased bile acid neosynthesis, suggesting the prebiotic potential of resveratrol [63].

### 3.2. Uremic Toxins

Uremic toxins are metabolites derived by gut microbiota metabolism of amino acids. The aromatic amino acids in proteins (tyrosine, phenylalanine, and tryptophan) can be metabolized by the GM [64,65] and liver of the host [66] to toxins such as indoxyl sulfate, indoxyl glucuronide, indoleacetic acid, p-cresyl sulfate, p-cresyl glucuronide, phenyl sulfate, phenyl glucuronide, phenylacetic acid, and hippuric acid. Circulating nitrogen metabolites as urea and asymmetric dimethylarginine characterize both chronic kidney disease [67] and CVD [68]. In the clinic, serum indoxyl sulfate level is now considered a predictive biomarker of coronary atherosclerosis [69]. In addition, hemodialysis patients present high serum levels of protein-related uremic toxins [70]. 

Mechanistically, both platelets and blood vessel components are susceptible to uremic toxins. Indoxyl sulfate activates platelets, increasing their aggregation in response to thrombin and collagen [71]. In ECs, indoxyl sulfate activates NF-κB signaling, upregulating ICAM-1 and monocyte chemotactic protein-1 (MCP-1) [72]. In addition, indoxyl sulfate inhibits NO synthesis and up-regulates reactive oxygen species (ROS), thus contributing to endothelial dysfunction and atherosclerosis [73]. 

The antioxidants N-acetylcysteine and apocynin, inhibitors of NADPH oxidase, can mitigate the proapoptotic effect of p-cresyl sulfate [74]. In endothelium, p-cresyl sulfate increases TNF-α, MCP-1, ICAM, and VCAM expression, thereby promoting atherogenesis [75]. The metabolite has also been reported to induce endothelial dysfunction and apoptosis, which were reverted by caffeic acid, a polyphenol present in white wine able to restore NO production and reduce ROS [76] (Table 1).

Since p-cresyl sulfate is hard to eliminate by dialysis [77], its biosynthetic pathway seems to be a more reliable target of intervention, together with strategies able to ameliorate its deleterious effect on EC function and angiogenesis.

**Table 1:** Studies to control intestinal gut metabolites useful in endothelial dysfunction related cardiovascular diseases.

Disorder	Model	Metabolite(s)	Intervention	Prevalent Mechanism	Reference
Coronary artery disease	Mouse cells and tissue	TMAO	DMB	Inhibition of foam cell formation	[56]
			FMO3 silencing or inhibition		[78]
			Resveratrol		[63]
	Mouse tissue	-	Probiotics	Reduce vascular inflammation	[79]
Hypertension	Mouse	SCFA	SCFA	Activation of G-protein coupled receptor-41	[45,80]
	Mouse tissue	SCFA	High-fiber diet	Increased SCFA-producing bacteria	[81,82,83,84,85,86]
	Human hypertensive patients	-	Dietary fibers, probiotics	Regulation of renin–angiotensin system.	[86,87,88,89,90]
	Human hypertensive patients	-	Minocycline, Vancomycin	Increased the diversity of GM and reduced Firmicutes	[36,91]
Hypertension and endothelial dysfunction	Spontaneous hypertensive rats (SHR)	-	Gallic acid	Angiotensin converting enzyme inhibition	[92]
Endothelial dysfunction	Cultured cells	Uremic toxins	Caffeic acid	Increased NO production and reduced ROS	[76]

### 3.3. Peptides

Bacteria release proteins and peptides that function on other microbes and the rest of the body. Pathological bacteria can release peptides which disrupt the gut–blood barrier, worsening the inflammatory status of the intestine and allowing the diffusion of bacteria into the circulation. Examples are peptides which act as ligands specific for Toll-like receptor in microvascular intestinal endothelial cells, where they increase permeability, transmigration, and angiogenesis [93].

Moreover, bacteria communicate with each other during gut colonization. A tight balance of cell-to-cell signaling maintains the homeostasis of microbial ecosystem. This includes peptides, which function either as antimicrobials to control neighboring bacteria, or as quorum-sensing peptides to actively colonize mucosa and form biofilms. A cross-talk between gut bacteria and cancer cells has been recently reported by means of circulating quorum-sensing peptides. In co-culture assay, the incubation of colon cancer cells with specific quorum-sensing peptides has been associated with metastasis through the upregulation of various angiogenic factors such as VEGF, hepatocyte growth factor, IL-6, and stromal-cell-derived growth factor [94].

### 3.4. Short-Chain Fatty Acids (SCFAs)

SFCAs are known beneficial metabolites for blood vessel control. SCFAs are fatty acids with fewer than six carbon atoms, including the most abundant, acetic acid, propionic acid, butyric acid, and the less abundant valeric acid and caproic acid. As previously mentioned, SCFAs are the result of bacterial fermentation of undigested carbohydrates, mainly carried out by *Lactobacillus* and *Bifidobacterium*. The most abundant SCFA in the colon, acetic acid, derives from carbohydrate fermentation, or can be synthesized from hydrogen and carbon dioxide or formic acid [95,96]. Three distinct pathways, including the succinate pathway, acrylate pathway, and propanodiol pathway [97], can generate propionic acid. Bacteria produce butyric acid by two different pathways: One converts butyryl-CoA into butyrate using phosphotransbutyrylase and butyrate kinase (e.g., *Coprococcus* species) [98,99], and the other converts butyryl-CoA in butyric acid by butyryl-CoA/acetate CoA-transferase (e.g., *Faecalibacterium*, *Eubacterium*, and *Roseburia*) [100].

Bacteria synthetize SCFAs in sequential steps from glycolysis of glucose to pyruvate, to acetyl-CoA, and finally to acetic acid, propionic acid, and butyric acid. Alternatively, amino acids can be used for SCFA biosynthesis. Glucose and amino acids are digested, respectively, from starch and protein in the small intestine and are rapidly absorbed into the circulation. Thus, dietary fibers are the main substrates used to produce SCFAs by intestinal microbes. Both inulin, a plant-derived fructan, and guar gum are prebiotic fibers [82,83,84]. Beside inulin-induced loss of weight, many clinical trials have confirmed the health benefits of inulin in various disorders [85]. Additionally, dietary fiber can selectively increase the abundance of SCFA-producing bacteria [86].

Hypertension has been associated with decreased gut microbial diversity and SCFA-producing bacteria and an increase in lactic acid producing bacteria [36,101]. Additionally, the abundance of SCFA-producing bacteria in obese pregnant women is negatively correlated with blood pressure in early pregnancy [102]. Taken together, these findings document the importance of probiotic and prebiotic supplementation in the early stages of pregnancy, and in general in adult/elderly individuals, to normalize blood pressure. 

Sodium butyrate has been extensively evaluated for its effects on angiogenesis. While its anti-angiogenic effects were largely established in tumors via histone deacetylase inhibition at high concentrations [103,104], low concentrations of sodium butyrate have been reported to stimulate angiogenesis in wound-healing models. The mechanisms comprise upregulation of VEGFR and the post-receptor signaling enzymes [105].

From a mechanistic point of view, SCFAs have been demonstrated to bind to defined G-protein coupled receptors (GPCRs) 41, 43, 109a, and olfactory receptor (OLFR) 78 in mice (homologous with Olfr59 in rats) to trigger intracellular signaling in various cell types [106]. OLFR78 has been found to be expressed in blood vessels and is activated by acetic acid and propionic acid (but not by butyric acid) to modulate blood pressure [80,107,108]. 

Nevertheless, it is highly accepted that SCFAs play important roles in human health (Table 1), and the widespread use of prebiotics, now widely present in the market, reinforces this finding.

### 3.5. Gaseous Metabolites

Gut flora produce a range of gaseous molecules. In the mammalian colon, H_2_S is one of the main gaseous transmitters released by sulfate-reducing bacteria. Other gut-bacteria-derived volatile compounds that affect the circulatory system are nitric oxide (NO), carbon monoxide (CO), and methane.

#### 3.5.1. Hydrogen Sulfide (H_2_S)

A number of studies from our lab have shown that H_2_S is an important regulator of the circulatory system [109]. Up to now, research has usually been focused on the effects of the H_2_S produced by cardiovascular tissue enzymes. However, some recent evidence suggests control of arterial blood pressure by H_2_S released by the colon through the same cellular and molecular mechanisms [50].

Sulfate-reducing bacteria (SRB) are ubiquitous members of mammalian colon GM. *Desulfovibrio (D. piger, D. desulfuricans)*, *Desulfobacter*, *Desulfobulbus*, and *Desulfotomaculum* are the dominant bacteria, producing H_2_S from two substrates: a sulfate and an electron donor for the sulfate reduction. While SRB represent a nonenzymatic source of H_2_S, the enzymatic generation of H_2_S originates from either gut bacteria or colonic tissues. Several anaerobic bacterial strains (*E. coli, Salmonella enterica, Clostridia*, and *Enterobacter aerogenes*) convert cysteine to H_2_S, pyruvate, and ammonia via cysteine desulfhydrase. An additional reaction for H_2_S generation is sulfite reduction, which has been described in *E. coli*, *Salmonella*, *Enterobacter*, *Klebsiella*, *Bacillus*, *Staphylococcus*, *Corynebacterium*, and *Rhodococcus*.

Most pharmacological studies to date have been performed by administration of H_2_S donors that produce a decrease in arterial blood pressure due to vasodilation. The mechanisms behind H_2_S-mediated vasodilation include the opening of ATP-sensitive potassium channels, upregulation of eNOS, and inhibition of phosphodiesterase-5, thus prolonging NO intracellular activity. In addition to vessel-tone control, H_2_S has been demonstrated to exert cardioprotective, pro-angiogenic, and cytoprotective effects, while disturbances in H_2_S homeostasis have been proposed to influence the etiology of cardiovascular and metabolic diseases [110,111].

#### 3.5.2. Nitric Oxide (NO)

The probiotic strains *Lactobacillus* and *Bifidobacterium* participate in NO production by decreasing gut pH, which increases nonenzymatic nitrite reduction in the intestine [112]. In contrast, *Desulfovibrio vulgaris* converts NO to nitrates [113]. For the protective effect of NO (mainly derived from eNOS) on all the players of cardiovascular system and on angiogenesis control see References [114,115], among others. 

NO, however, along with H_2_S, has been described as a double-edged sword mediator. It is protective at nanomolar concentrations such as the ones synthetized by eNOS, but micromolar concentrations are cytotoxic to cells and microorganisms. High NO concentrations derive from induction of the inducible isoform iNOS. One of the major determinants of iNOS upregulation is bacteria-derived lipopolysaccharide (LPS), shed by Gram-negative bacteria such as *E. coli*. iNOS-derived NO reacts with oxygen radicals, giving rise to highly reactive peroxynitrates which worsen infection-associated pathologies such as septic shock, characterized by blood pressure fall [116].

#### 3.5.3. Carbon Monoxide (CO)

The enzyme heme oxygenase (HO) catalyzes the degradation of heme, with production of biliverdin, ferrous iron, and CO. Inducible HO (HO-1) and constitutive HO (HO-2) are involved in endogenous CO synthesis in mammalian tissues, HO-1 being mainly expressed in the gastrointestinal mucosa. In addition, GM (*E. coli*) have been reported to express HO homologs [117]. Analogous to NO and H_2_S, CO has been found to exert vasorelaxation and cardiac protective effects [118,119]. The potential properties of gut-derived CO on systemic circulation, however, remain to be clarified.

Moreover, the situation is complicated by the fact that the three gaseous transmitters mentioned above and their biochemical signaling cascades interact at different levels [120,121].

### 3.6. Metabolites Deriving from Gut Metabolism of Xenobiotics

It is now well established the human gut microbiome plays a prominent role in xenobiotic transformation, even if most of the genes and enzymes responsible for this metabolism are unknown. Examples of xenobiotics of which the metabolism then affects the cardiovascular system are diet components such as polyphenols. Polyphenols are a large group of biologically active molecules produced by plants and algae. Their effects on animals are multiple, and dependent on the intake dose and the circulating metabolites [32,33,122]. The list of plant-derived molecules also includes the toxins which at defined doses are used as cardiac drugs, such as digoxin. Its metabolism by the actinobacterium *Eggerthella lenta* is responsible for animal protection in cases of intoxication and altered bioavailability when used as a drug, thus emphasizing the need for personalized dosing [123,124].

#### 3.6.1. Anthocyanins

Anthocyanins are glycosyl-anthocyanidins widely distributed in plants, with color depending on pH. Anthocyanidins are flavonoids with different functional groups covalently linked to the three cycles. Anthocyanins’ beneficial effects have been demonstrated in various pathological conditions including obesity and diabetes, CVD, cancer, and visual and brain functional impairment [125,126]. Mechanistically, the positive properties of anthocyanins on cardiovascular wellness include antiplatelet activity, vasorelaxation due to increased NO production via eNOS upregulation, protection of cardiac cells from ROS-induced apoptosis, and increased high density lipoproteins (HDL) cholesterol [127,128,129,130]. 

Further studies have confirmed that some GM anthocyanin metabolites are responsible for the beneficial effect on atherosclerosis. Ingested dietary anthocyanins are partially absorbed, while large amounts pass into the colon where they are degraded by GM to free anthocyanidins and protocatechuic acid [131]. One of the microbiotic anthocyanin metabolites is gallic acid, which has been shown to increase NO levels through phosphorylation of eNOS [132] and to inhibit angiotensin-converting enzyme (comparably to the drug captopril), significantly reducing blood pressure in spontaneously hypertensive rats, an experimental model of human essential hypertension [92] (Table 1).

Interestingly, anthocyanins can also modulate the GM composition. For example, malvidin-3-glucoside can enhance the proliferation of some beneficial bacteria such as *Bifidobaterium* spp. and *Lactobacillus* spp. [133]. On the other hand, gallic acid can decrease some potentially harmful bacteria such as *Clostridium histolyticum*, with no effect on advantageous bacteria [133].

Thus, anthocyanins play a pivotal role in normalizing the altered microbiota taxonomic composition in CVD patients. These results suggest that anthocyanins isolated from vegetables, algae, and fruits exert multiple therapeutic effects and show a great potential as CVD interventions.

#### 3.6.2. Phytoestrogens

Phytoestrogens are compounds with controversial activity on mammal cells. Phytoestrogens are chemically similar to human estrogens and include isoflavones, ellagitannins, and lignans. In the gut, phytoestrogens can be further processed to more active compounds, such as equol, O-desmethylangolensin, dihydrodaidzein, dihydrogenistein, enterolactone, and enterodiol [134,135,136]. The enterolactone and enterodiol biosynthetic pathways have been found in several bacterial strains metabolizing lignans [137]. Lignans are degraded from lignin, an abundant plant-derived polymer, secondary to cellulose in amount in the Earth, by GM [138,139]. Furthermore, several bacterial strains present in the gut, such as *Adlercreutzia equolifaciens*, *Eggerthella sp*. YY7918, *Lactococcus garvieae*, *Slackia equolifaciens*, *Slackia isoflavoniconvertens*, and *Slackia sp*. NATTS can metabolize daidzein to equol and O-desmethylangolensin [140,141,142,143].

Phytoestrogens have been reported both to reduce the risk and progression of various cancer types and to protect the cardiovascular system via different molecular mechanisms, which involve both estrogen receptor occupancy and other molecular pathways. In both cases, the antioxidant and anti-inflammatory potential of the phytoestrogens has been implicated in health promotion [144]. Accordingly, urinary total and individual phytoestrogen levels were significantly inversely correlated with the serum inflammatory biomarker C-reactive protein [145]. Additionally, one report showed that high serum enterolactone level was linked to reduced CVD mortality [138], while low serum enterolactone was associated with augmented lipid peroxidation, monitored by F2-isoprostane plasmatic concentration [137]. 

Phytoestrogens can bind to estrogen receptors, working as agonists or antagonists [146,147]. Thus, the effects of phytoestrogens can be biphasic: they can either increase vasodilation through NO production, or exert some prothrombotic or proinflammatory effects [148]. 

Based on the above findings, the topic is highly debated and research is ongoing to elucidate the proper composition and formulation of these very active compounds to be used in cardiovascular or metabolic disorders [32,33].

## 4. Conclusions and Perspectives

In summary, microbiome analyses have resulted in several newly identified metabolites which exert defined functional roles in the control of the cardiovascular system and angiogenesis [52]. The list provided in this review is partial, and the number of these derivatives, as well as their known functional properties, is growing. These findings show great promise for the identification of novel therapeutic targets in the microbial biosynthetic pathways for several human diseases, including CVD and endothelium-dependent disorders. Of course, the safe use of these novel products will require a thorough understanding of how they interact with the host, both in normal conditions and pathological states; this may be achieved by combining meta-omics approaches and functional testing utilizing germ-free mice, in vitro co-culture systems, and intestinal organoid technologies.

Meanwhile, the approaches of re-establishing or editing a “healthy” microbiota have included the use of probiotics (or prebiotic and symbiotic), special diet components (high fiber consumption), and fecal microbiota transplant [149]. Obviously, these approaches have to be proposed together with diet control and regular physical activity, widely recognized to reduce risk of CVD and other diseases.

Several probiotics have been evaluated in clinical trials in relation to blood pressure regulation. The probiotic *Lactobacillus* has been proven helpful in controlling blood pressure by protecting endothelial function and reducing vascular inflammation [150]. A meta-analysis of nine randomized trials including 543 participants showed that a daily dose of >10^11^ CFU of *Lactobacillus helveticus* prevented hypertension or controlled blood pressure by regulating the renin–angiotensin system [89]. These studies suggest that GM plays an important role in the control of blood pressure and cardiovascular homeostasis, and that the correction of gut dysbiosis by probiotics may be beneficial in CVD. 

Transplantation of fecal microbiota, which consists in the transfer of intestinal flora from a donor to the intestinal tract of a recipient, aims to increase the diversity of the gut microbiota and change its composition. While it has been successful in treating inflammatory bowel disease and *Clostridium difficile* infection [151,152], the risks of spreading unknown microorganisms to the recipient still remain [153]. We are in the early days of this new treatment, and only appropriately designed clinical trials and post-marketing surveillance will reveal its safety in the long term.

The editing of GM may, however, have advantages, mainly in offering the possibility of relatively non-invasive and inexpensive therapeutics to a large number of people worldwide. Another intriguing application of these findings is related to astronauts’ recovery from the body dysregulation caused by long-term residence in a space environment. Interestingly, their conditions mimic those of aged/disabled people and frail patients under extreme/critical medical conditions (such as radiation exposure). Further research on these subjects will allow the results obtained from this limited human sample to be applied to a larger population. 

A critical aspect that has only partially been examined in the clinic is the effect of sex and of particular conditions such as pregnancy on gut composition, and therefore on cardiovascular function and risk of developing pathologies [154,155]. Today, preeclampsia remains a medical condition critical for both the mother and the fetus, and gut microbiota could be an ideal substrate for the definition of both biomarkers and therapeutic corrections [156].

In conclusion, in designing various approaches for normalizing gut composition or readdressing microbial metabolism, researchers and medical doctors should consider the complex interplay among the microbiota, immune, cardiovascular, endocrine, and nervous systems, which is currently far from being completely elucidated.
